# Effects of Serine/Arginine Enriched Protein BmUP on the Development of Male Silkworm Reproductive Organs

**DOI:** 10.3390/cimb44020061

**Published:** 2022-02-14

**Authors:** Chun-Bing Chen, Juan Li, Xuan Deng, Lian-Lian Liu, Jing Deng, Xing-Fu Zha

**Affiliations:** 1 State Key Laboratory of Silkworm Genome Biology, Institute of Sericulture and Systems Biology, College of Sericulture, Textile and Biomass Sciences, Southwest University, Chongqing 400715, China; chenchunbing@email.swu.edu.cn (C.-B.C.); worker@swu.edu.cn (J.L.); dx1241786053@email.swu.edu.cn (X.D.); l23456@email.swu.edu.cn (L.-L.L.); d15700540673@email.swu.edu.cn (J.D.); 2 School of Life Sciences, Southwest University, Chongqing 400715, China

**Keywords:** serine/arginine-rich, CRISPR/Cas9, transgene overexpression, BmUP, *Bombyx mori*

## Abstract

Serine/arginine-rich proteins are a class of highly conserved splicing factor proteins involved in constitutive and alternative splicing. We screened a low molecular weight serine/arginine rich protein from silkworms and named it BmUP. Temporal and spatial expression analysis indicated that the *BmUP* gene was specifically expressed in the silkworm testis, and the highest expression occurred in the pre-pupa stage from the fifth instar to the moth stages. Here, we generated *BmUP* knockout individuals with the CRISPR/Cas9 system. Both the internal and external genitalia of knockout individuals were abnormal in knockout compared with wild-type male silkworms. In transgenic silkworms overexpressing *BmUP*, male silkworms showed a phenotype similar to that of the knockout individuals, whereas female individuals showed no significant differences from the wild type. In addition, by conducting promoter analysis, we identified *Bmachi*, a transcription factor that regulates the *BmUP* gene. Gel migration experiments revealed that BmAchi specifically binds the *BmUP* promoter. Quantitative real-time PCR showed that an increase in *Bmachi* expression up-regulated the expression of *BmUP*. In contrast, when the expression of *Bmachi* decreased, the expression of *BmUP* also downregulated in the experimental group compared with the control group. These results provide new insights for studying the effects of serine/arginine-rich proteins on the development of silkworm genitals.

## 1. Introduction

In most higher eukaryotes, splicing of precursor RNA is an essential step in the expression of protein coding genes. Alternative splicing is a common phenomenon in gene expression and is one reason for the diversity of protein structure and function in the biological world [1,2]. For example, tens of thousands of genes in the human genome can produce hundreds of thousands of proteins or more, and approximately 92–94% of genes undergo alternative splicing [3]. The splicing process involves a large molecular complex that removes introns from the precursor RNA to form mature RNA. In this complex, the spliceosome is composed of nucleoprotein particles and many other proteins [4]. In the spliceosome complex, serine/arginine-rich proteins, such as U2AF35, U2AF65 and U1-70K, have important roles [5]. In addition to being a component of the spliceosome, SR proteins can act as hydrolase or helicase enzymes and are involved in conformational changes in protein molecules [6]. SR proteins are a family of serine/arginine-rich proteins characterized by their RS structural domain. The discovery of these proteins arose from the identification of Drosophila splicing factors SWAP (suppressor of white-apricot) [7], Tra (transformer) [8] and Tra-2 (transformer-2) [9]. The first identified SR proteins were SF2/ASF (ARSF1) and SC35 (ARSF2), which were derived from human cell lines and have similar sequence characteristics to those of the Drosophila splicing regulator [10,11].

SR proteins are RNA binding proteins with important roles in RNA splicing. Classical SR proteins are recognized by the monoclonal antibody mb104 and are mainly composed of one or two RNA recognition domains (RRM domain) at the N-terminus and a serine/arginine-rich RS domain at the C-terminus. The RS domain is composed of continuous RS or the SR repeat sequence [12]. The RNA recognition domain binds RNA. Although binding is not strong, it has specificity. RS structural domains mainly mediate protein–protein reactions and can also act as nuclear localization signals [13]. Several RS structural domains influence RNA binding interactions [14]. Due to the differences in structural domains or the lack of RRM, this class of proteins is also referred to as SR-related or SR-like proteins. SR proteins play important roles in mRNA metabolism. In addition to participating in the constitutive and alternative splicing of RNA, they participate in post-splicing regulatory processes, such as mRNA nuclear export, nonsense-mediated mRNA decay and mRNA translation [15,16]. In addition, SR proteins have a role in maintaining the stability of the genome. Removing SC35 from mouse embryonic fibroblasts causes severe double-stranded DNA breaks [17]. Inactivation of SF2/ASF from chicken DT40 cells also induces severe double-stranded DNA breaks and DNA recombination, thus indicating that SR protein loss induces cell cycle arrest and apoptosis [18]. Simultaneously, SR proteins play important roles in organizing nuclear gene networks and maintaining cell cycle progression [19]. SR proteins not only are important in the gene expression process but also are key factors in the occurrence of several diseases. Abnormal expression may occur in some tumors. An SR protein is closely associated with the occurrence of many human diseases. Spinal muscular atrophy caused by SMN protein deficiency is a genetic cause of death in children. Wee, CD et al. have found that knocking down SRSF2 or SRSF3 in a cell line from patients with spinal muscular atrophy enhances SMN protein. Studies are providing new ideas for the treatment of spinal muscular atrophy [20]. Researchers have transfected SRSF7 specific small RNAs into colon cancer cell lines (HCT116) and lung cancer cell lines (A549) and found that knockdown of the SRSF7 gene effectively inhibits the proliferation of cancer cells and promotes cell apoptosis [21]. As a member of the SR protein family, SRSF6 has been found to be upregulated in colorectal cancer samples. Through a series of in vivo and in vitro experiments, SRSF6 has been found to promote the proliferation and metastasis of colorectal cancer cells [22].

In eukaryotes, sex determination is an important process controlling the development of male or female sex. In sex determination in Drosophila, upstream sex lethal (*sxl*), transformer (*tra*) and transformer-2 (*tra2*) are key genes that activate *dsx* for female-specific splicing [23]. In Bombyx mori, an economically important insect, the sex determination mechanism is quite different from that in Drosophila due to the absence of the *tra* gene. Studies have shown that the regulation mechanism of Bombyx mori *dsx* remains unclear [24]. Due to the fact that regulatory factors TRA and TRA2, which are involved in sex determination in Drosophila, belong to the SR protein family, we suspected that similar splicing regulators might also function in sex regulation in silkworms. In this study, we screened for an SR protein in silkworms and named the identified protein BmUP. We knocked out the *BmUP* gene by using CRISPR/Cas9 technology and constructed transgenic silkworms overexpressing *BmUP*. By performing these two techniques, we demonstrated the role of the *BmUP* gene in silkworm sex regulation.

## 2. Materials and Methods

### 2.1. Insects and Cells

The non-diapause strain D9L of *Bombyx mori* has been deposited in the State Key Laboratory of *Bombyx mori* Genomic Biology, Southwest University, Chongqing, China. The eggs were kept at 25 °C and 75% relative humidity under 12 h light/12 h dark until hatching. Silkworm larvae were reared with fresh mulberry leaves. The *Bombyx mori* cell line BmE was derived from the silkworm embryos and cultured in Grace insect medium supplemented with 10% fetal bovine serum. BmN cells were derived from silkworm ovarian cells and cultured in TC100 medium. Cell culture temperature was maintained at 27 °C.

### 2.2. SgRNA Design and Synthesis

The gRNA was designed according to the coding sequence of the *BmUP* gene according to the website CCTop (https://cctop.cos.uni-heidelberg.de:8043/ (accessed on 17 September 2020)). ATAG was added to the F end of the designed sequence, and the reverse complement, AAAC, was added to the R primer. The primers were annealed and self-ligated and then cloned into the pUC57 vector. The vector contained a T7 promoter and tracrRNA sequence, which were transcribed and synthesized in vitro to generate sgRNA. The recombinant Cas9 protein was purchased from TaKaRa (Cat. No. Z2641N). The splicing efficiency of sgRNA was verified with in vitro splicing experiments. The target fragment containing gRNA sequence was amplified from the silkworm genome, mixed with Cas9 protein and gRNA and then subjected to restriction digestion for verification. The primers used in this article are shown in Table 1. We mixed 1 μg of Cas9 protein and 375 ng of each sgRNA in a volume of 5 μL and injected the Cas9/sgRNA mixtures individually into each silkworm egg within 2 h of egg laying. Successfully hatched individuals were reared to the moth stage, the genital phenotype was observed and then the genome was extracted to test knockout efficiency.

### 2.3. Vector Construction

The full-length coding sequence of the *BmUP* gene was cloned from silkworm testis. The forward primer had a BamHI restriction site and a Flag-tag sequence, and the reverse primer had a NotI site. After BamHI and NotI digestion, the PCR product was inserted into the pSL1180 (4-EGFP-SV40) vector to produce a recombinant pSL1180-BmUP vector. The pSL1180-BmUP vector was digested with AscI and ScaI and then cloned into the piggyBac (3xP3-Red-SV40) vector. By performing sequencing, we confirmed that the piggyBac-BmUP (A4-*BmUP*-SV40-3xP3-Red-SV40) vector was obtained.

### 2.4. Construction of BmUP Overexpressing Transgenic Line

The recombinant piggyBac-BmUP vector plasmid and the helper plasmid (pHA3PIG helper) were mixed at a volume ratio of 1:1 and then injected individually into each silkworm egg within 2 h of laying. The temperature was kept at 25 °C, and humidity was controlled until the silkworm eggs successfully hatched. The hatched G0 generation individuals were raised to moth stage and mated to produce G1 generation individuals. G1 individuals in the embryonic stage were screened with a fluorescence microscope (Olympus, Tokyo, Japan), and those with red fluorescence in the eye position were considered positive. The genomic DNA of transgenic individuals was extracted, digested with HaeIII enzyme overnight and self-ligated; reverse PCR was then performed to detect the insertion site in transgenic individuals.

### 2.5. Quantitative Real-Time PCR

According to the manufacturer’s instructions (Total RNA Kit., OMEGA, Biel, Switzerland), we extracted total RNA from each tissue of day-3 fifth-instar silkworms, reverse transcribed the RNA with a reverse transcription kit (Cat. No. RR047A) and adjusted the reverse transcribed cDNA template to 200 ng/ μL. Quantitative PCR was performed on an ABI7500 Real-Time PCR machine (Applied Biosystems, Foster City, CA, USA), and the SYBR^®^ Premix Ex Tag™ kit was used to perform the reactions. mRNA expression was calculated according to the Ct value. The primers used in the qPCR reaction are listed in Table 1. Silkworm sw22934 was used as an internal reference gene, and the experiment was performed with three biological and technical replicates.

### 2.6. Western Blot

The female transgenic and wild-type *Bombyx mori* moths were ground with liquid nitrogen, and protein was extracted on ice with NP40 lysis buffer supplemented with protease inhibitors (1:100 ratio). The supernatant was collected by centrifugation (4 °C, at 12,500 rpm for 20 min). A BCA kit (Beyotime, Shanghai, China) was used to detect the protein concentration, and the sample was heated with 5× protein loading buffer (containing β-mercaptoethanol) at 98 °C for 10 min. SDS-PAGE was performed with a 12.5% protein gel (concentrating gel run with 100 volts, separating gel run with 120 volts), and the target protein was transferred to a PVDF membrane (25 volts, 20 min) with the semi-dry transfer method. TBST prepared with 5% skimmed milk powder was used for blocking at room temperature for 1 h. The membrane was incubated with anti-Flag-tag (Beyotime, Shanghai, China) at room temperature for 2 h, then with horseradish peroxidase labeled goat anti-mouse IgG secondary antibody. After 1 h incubation, the membrane was washed three times with TBST after each step for 10 min each. Finally, we used SuperSignal West Femto Maximum Sensitivity Substrate for exposure.

### 2.7. Subcellular Localization

The cells were spread into 12-well cell culture plates when cell density reached 80%, and pSL1180-Flag-BmUP and control plasmid were transfected into the cells, respectively. After 48 h, the culture medium was discarded, and the cells were fixed with 4% paraformaldehyde for 15 min after washing with PBS for 3 times. Immunofluorescence blocking solution (0.01 g BSA powder + 100 μL sheep serum + 10 μL Tritonx-100 + 890 μL 1 × PBS) was prepared during fixation, and the cells were washed 3 times with PBS after 1 h of blocking and then incubated with immunofluorescence primary antibody hybridization dilution (0.01 g BSA powder + 10 μL Tritonx-100 + 990 μL 1 × PBS + 1 μL Flag-tag) for 1 h, and after incubation, PBS was washed 3 times before incubation with immunofluorescent secondary antibody hybridization dilution (0.01 g BSA powder + 10 μL Tritonx-100 + 990 μL 1 × PBS + 2 μL cy3-labeled secondary antibody) for 1.5 h. After incubation, PBS was washed 3 times before staining with DAPI for 10 min.

### 2.8. Dual Luciferase Reporter Experiment

According to the *BmUP* promoter, the transcription factor prediction was performed with the JASPAR (https://jaspar.genereg.net/ (accessed on 18 March 2021)) website, and the predicted transcription factor was cloned into the pSL1180 vector. The promoter was truncated according to the binding sites of the transcription factor and the promoter to 297 bp, 421 bp, 937 bp and 1939 bp lengths. The promoters of different lengths were amplified from the silkworm genome and cloned into the T5-zero vector (TRAN, Shanghai, China). The vector was digested with XhoI and HindIII to obtain the target fragment with restriction enzyme sites. The dual luciferase reporter PGL3 (IE1-SV40) vector was digested with XhoI and HindIII and then ligated to the target fragment. PrL (Vg-SV40) was used as an internal reference carrier. When BmE cells were cultured to a density of approximately 80%, they were seeded into a 24-well cell culture plate. After 24 h, PrL vectors and PGL3 vectors with different promoter lengths, and vectors for overexpression of transcription factors were co-transfected into BmE cells. After 48 h, we tested the fluorescence activity with a dual luciferase reporter kit (Promega, Madison, WI, USA).

### 2.9. Electrophoretic Mobility Shift Assays

Gel migration experiments can be used to verify whether transcription factors interact with promoters. We first constructed an Myc-tagged *Bmachi* overexpression vector pSL1180-*Bmachi*-Myc and then synthesized biotin-labeled probes based on the three predicted binding sites. We transfected pSL1180-*Bmachi*-Myc and pSL1180-EGFP-Myc plasmids into BmE cells and then extracted nucleoproteins 48 h after transfection and detected successful protein overexpression by Western blotting. The binding of protein and nucleic acid was verified with chemiluminescence kit (Beyotime, Shanghai, China).

### 2.10. Data Analysis

Data analysis and mapping were performed in the GraphPad Prism 5 software. All data are expressed as the mean ± standard deviation. An unpaired two-tailed Student’s *t*-test was used to determine statistical significance. *p* < 0.05 was considered to indicate a significant difference (* *p* < 0.05, ** *p* < 0.01 and *** *p* < 0.001).

## 3. Results

### 3.1. BmUP Is Highly Expressed in the Testis

**Figure 1:**
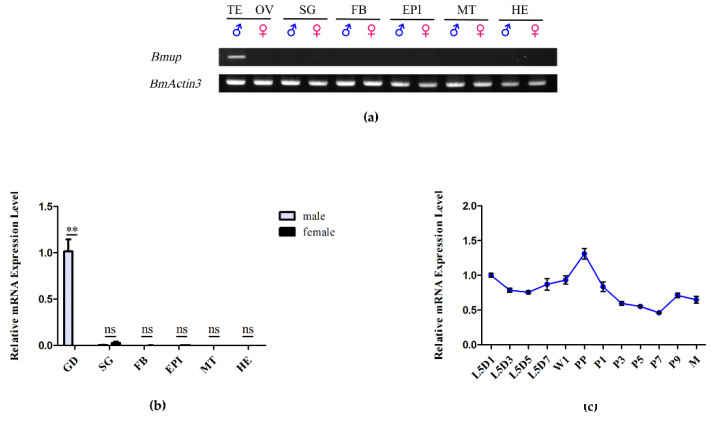
Expression pattern of the *BmUP* gene. (**a**) Semi-quantitative analysis of *BmUP* gene expression in each tissue of fifth-instar, 3-day-old silkworms. BmActin3 is an internal reference gene. TE, testis; OV, ovary; SG, silk gland; FB, fat body; EPI, epidermis; MT, malpighian tubule; HE, head. (**b**) Q-RT-PCR analysis of the transcript abundance of *BmUP* in larvae at the third day of the fifth instar. GD, gonad (** *p* < 0.01, ns indicates no significance). (**c**) The expression pattern of the *BmUP* gene in the testis from the first day of the fifth instar to the moth stage. L5D1, the first day of the fifth instar; L5D3, the third day of the fifth instar; L5D5, the fifth day of the 5th instar; L5D7, the seventh day of the 5th instar; W1, wandering; PP, pre-pupa stage; P1, the first day of the pupa stage; P3, the third day of the pupa stage; P5, the fifth day of the pupa stage; P7, the seventh day of the pupa stage; P9, the ninth day of the pupa stage; M, moth.

No prior research has described the relative expression of the *BmUP* gene in silkworms. To understand the temporal and spatial expression patterns of the *BmUP* gene in detail, we used semi-quantitative PCR and real-time fluorescence quantitative PCR to analyze the expression of *BmUP* in the gonads, silk glands, fat bodies, epidermis, Malpighian tubes and heads of female and male silkworms. We collected different tissues on the third day of the fifth instar by dissection. RT-PCR and qPCR showed that the *BmUP* gene is expressed almost exclusively in silkworm testis tissues (Figure 1a,b). Thus, the *BmUP* gene might play an important role in the development of the silkworm testis. We then mapped the expression profile of the *BmUP* gene from the fifth instar to moth stages. The gene expression peaked in the pre-pupa stage (Figure 1c). Due to the fact that, in mature silkworms, sperm cells gradually become mature sperm, we speculated that this gene might be involved in sperm development and maturation.

### 3.2. CRISPR/Cas9-Mediated Mutagenesis of the BmUP Gene

**Figure 2:**
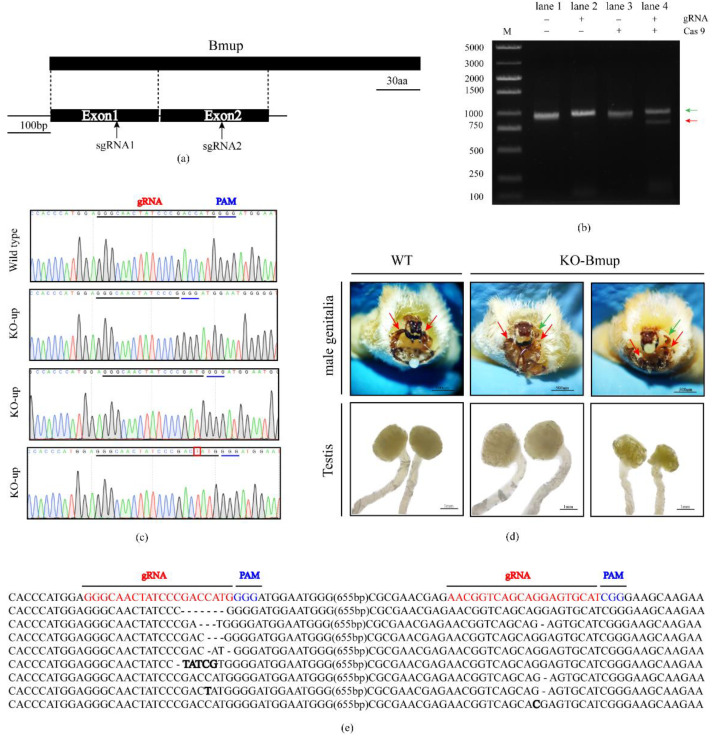
CRISPR/Cas9 mediated mutagenesis of *BmUP*. (**a**) Locations of sgRNAs relative to the *BmUP* gene. (**b**) Analysis of cleavage products by gel electrophoresis. Lane1, untreated target fragment; lane2, target fragment treated with sgRNA; lane 3, target fragment treated with Cas9 protein; lane 4, target fragment treated with Cas9/sgRNA mixture. The red arrowhead indicates cleaved fragments, and the green arrowhead indicates uncleaved fragments. (**c**) Chromatograms of DNA sequences of wild-type and knockout individuals. The gRNA target site is underlined, and the inserted base is indicated by a red rectangle. (**d**) Phenotype and gonad observation in knockout individuals. WT represents wild-type and KO-up represents knockout individuals. The red arrow represents the clasper, and the green arrow represents hyperplastic tissue. (**e**) Mutation sequences at the target sites of *BmUP* induced by CRISPR/Cas9. The wild-type sequence is shown on the first line, the sequences of sgRNA-targeted sites are in red and the protospacer adjacent motif (PAM) sequences are shown. Dotted lines represent missing bases, and bold letters represent alternative bases.

In order to investigate whether the *BmUP* gene might be involved in the development of the silkworm testis, we used the CRISPR/Cas9 system to knock out the *BmUP* gene. We designed two sgRNAs to target the first and second exons of the *BmUP* gene, cloned them into the Puc57 vector and then synthesized mature gRNA through in vitro transcription (Figure 2a). Before editing, we performed an in vitro assay to assess the editing efficiency of the synthesized gRNAs. We cloned a fragment containing two gRNAs from the silkworm genome. We then mixed gRNA, Cas9 protein and genome fragments in a restriction enzyme digestion. The experimental group yielded smaller fragments than the target genome (Figure 2b). We then mixed the effective gRNA with Cas9 protein and injected them into silkworm eggs within 2 h of egg laying. Subsequently, we raised the successfully hatched eggs to the moth stage. We observed the internal and external genitalia of male moths and found that, the external genitalia of the experimental moths, compared with the wild-type moths, proliferated with rod-shaped or clasper-like tissues, and the original left-right symmetrical claspers were shifted down on one side and, consequently, could not hook the female moths during mating and allow for successful mating (Figure 2d). Moreover, we dissected internal genitalia and found that the left and right testes in the same silkworm had different sizes. To determine whether the abnormalities in the external genitalia were caused by knockout of the *BmUP* gene, we performed genomic testing on the abnormal individuals and cloned the fragment containing both gRNAs for sequencing (Figure 2c). We detected various editing forms at the position of the gRNA (Figure 2e).

### 3.3. Construction of a BmUP Overexpressing Transgenic Line

**Figure 3:**
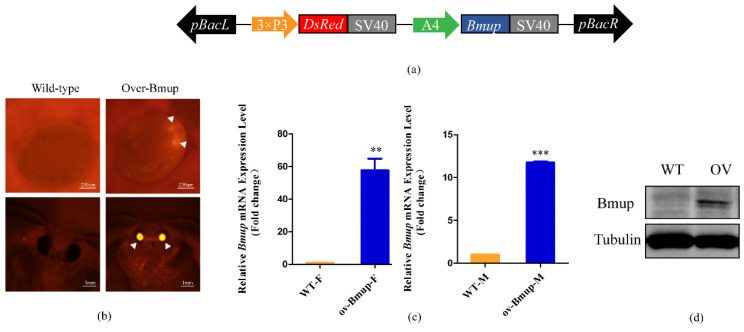
Establishment of Over-BmUP transgenic strains. (**a**) The transgenic expression vector pBac[3 xp3-DsRed, A4-BmUP-SV40] was constructed to overexpress Bombyx mori *BmUP*. The promoters were 3 xp3 and A4, and SV40 was used to stop transcription. DsRed is a Red Fluorescent Protein. (**b**) Fluorescence images of G1 eggs and moths for transgenic *BmUP* overexpression and wild type (WT) strains. The signal of DsRed in the transgenic strain is indicated with white triangles. (**c**) Relative mRNA levels of *BmUP* were investigated by qRT-PCR in the testis and ovaries in overexpressing individuals and wild-type individuals (*** *p* < 0.001, ** *p* < 0.01). (**d**) Immunoblot analysis of BmUP according to the Flag-tag.

In order to fully study the function of the *BmUP* gene in silkworms, we constructed a *BmUP* overexpression vector piggyback-BmUP (Figure 3a), which was mixed with the helper plasmid pHA3PIG and injected into 455 silkworm eggs within 2 h after egg laying. A total of 68 eggs hatched successfully and developed into adults. The injected silkworm eggs were raised to moth stage, and the moths were allowed to mate and lay eggs. At 6 or 7 days after egg laying, we performed fluorescence screening. Individuals with red fluorescence in their eyes were considered positive and were denoted Over-BmUP (Figure 3b). In order to detect the insertion position of the overexpression vector in the silkworm genome, we performed reverse PCR detection on the positive individual moths of G1 generation; cloned and sequenced the PCR products; and compared the results with the silkworm genome. The insertion site of transgenic Over-BmUP was found to be located in the intergenic region of chromosome 10 (Table 2).

By conducting real-time quantitative PCR, we analyzed the expression of the *BmUP* gene in male and female overexpressing individuals at the mRNA level. The testes and ovaries of transgenic individuals were collected for testing. The mRNA levels of *BmUP* were 60 times higher in transgenic than wild-type, and the expression in males was also approximately 10 times higher than that in the wild type (Figure 3c). The transgenic line Over-BmUP was successfully generated in both males and females. Tubulin was used as a control.

In order to further determine whether the Flag-tagged *BmUP* gene led to successful protein synthesis in transgenic individuals, we extracted proteins from wild-type and overexpressing moths and performed western blot detection. We observed clear bands for individuals with overexpression, but not wild-type (Figure 3d). Thus, BmUP protein was successfully expressed.

### 3.4. Cellular Location of the BmUP Gene and Observation of the Transgenic Phenotype

**Figure 4:**
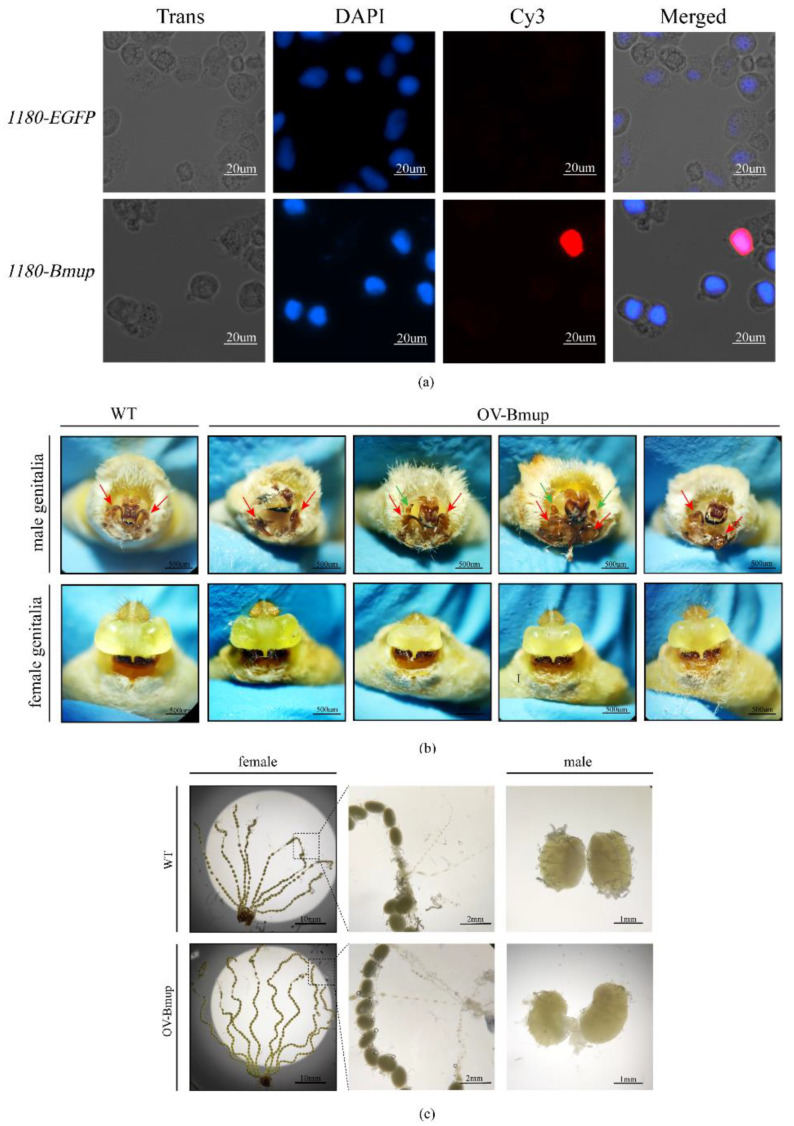
Cellular location of the *BmUP* gene and observation of the phenotype of overexpressing silkworms. (**a**) Subcellular location of *BmUP* in the silkworm cell lines (BmN) and pSL1180-EGFP treated cells as a control. DAPI-treated nuclei, red fluorescence for Cy3-treated UP protein and merged images are shown. (**b**) Observation of external genitalia in Over-BmUP transgenic strains. WT represents wild-type, and OV-BmUP represents overexpressing individuals. The red arrow represents the clasper, and the green arrow represents hyperplastic tissue. (**c**) Observation of the gonads. The female fallopian tube and the male testis is shown.

In order to determine the expression of the *BmUP* gene in cells, we constructed a recombinant vector, pSL1180-Flag-BmUP, for the overexpression of the gene in silkworm cell lines. We also constructed a control vector, pSL1180-EGFP, and performed immunofluorescence localization experiments in BmN cells. The BmUP protein was mainly localized in the nucleus and to a lesser extent in the cytoplasm (Figure 4a).

After feeding the transgenic positive individuals until the moth stage, we observed the internal and external genitalia of male and female moths. In a small number of transgenic male individuals, we found a phenotype similar to that of knockout individuals, with the production of redundant clasper-like tissue, a phenotype observed in three consecutive generations of positive individuals (Figure 4b). Anatomical observation of the internal genitalia revealed that the sizes of the left and right testes differed (Figure 4c). We observed no significant differences in external genitalia between female transgenic and wild-type individuals. We speculate that the reason why knockout individuals and overexpressing individuals had the same phenotype might have been that the change in *BmUP* gene expression affected the pathways controlling the development of the moth’s genitalia, thus indirectly affecting the changes in moth genitalia.

### 3.5. The Silkworm Meiosis Blocker Gene Achi/Vis Regulates the Expression of BmUP

**Figure 5:**
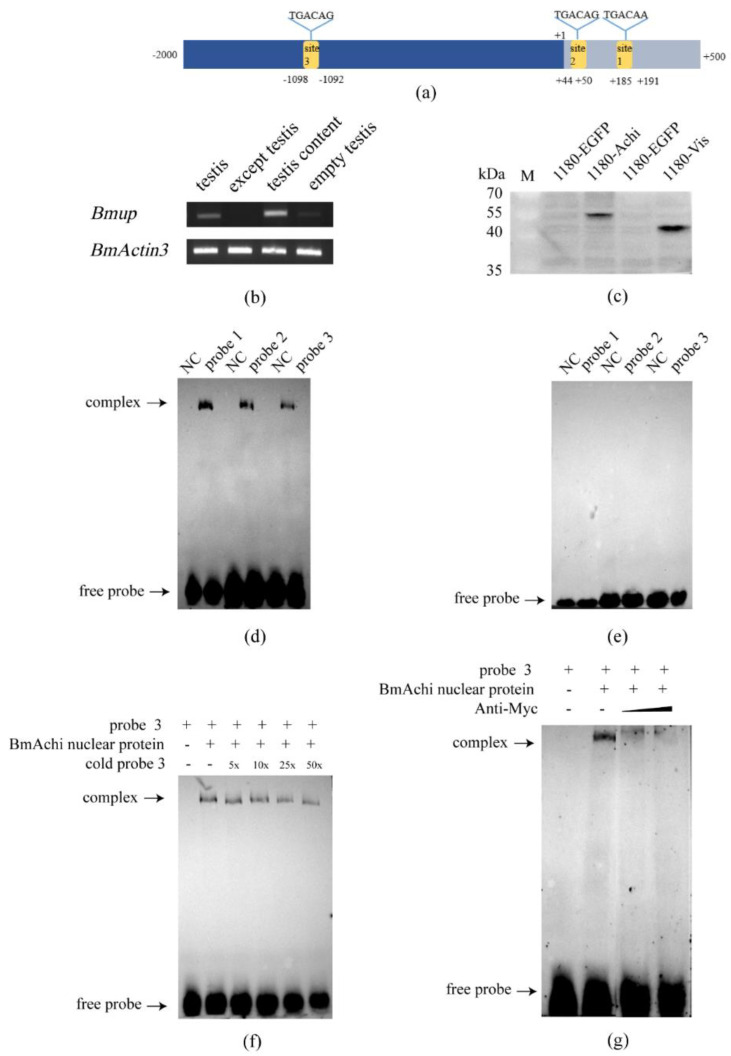
The binding of the transcription factor BmAchi and *BmUP* promoter. (**a**) Schematic diagram of the *BmUP* promoter. The yellow area represents the predicted binding site of the transcription factor BmAchi/BmVis, and the +1 position is the first base of *BmUP*. (**b**) Expression of *BmUP* in the testis; *BmActin3* is an internal reference gene. (**c**) Western blot detection of BmAchi and BmVis protein; pSL1180-EGFP is a control. (**d**,**e**) Combination experiment of the *BmUP* promoter with BmAchi and BmVis. NC indicates only biotin-labeled probes in the system but no transcription factor protein (lanes 1, 3 and 5). Probe 1, probe 2, and probe 3 represent the experimental groups in which the three probes react with nucleoprotein (lanes 2, 4 and 6). (**f**) EMSA cold competition experiment with the transcription factor BmAchi and probe 3. (**g**) EMSA super-shift experiment with the transcription factor BmAchi and probe 3.

**Figure 6:**
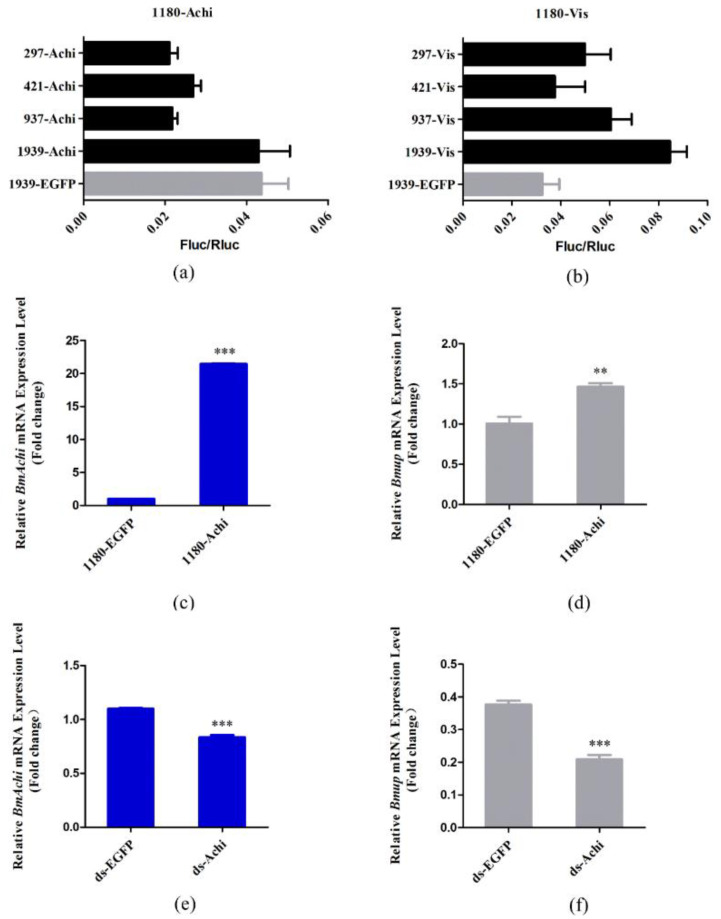
The regulatory effect of the transcription factor BmAchi on *BmUP*. (**a**,**b**) Dual luciferase reporter experiment on the transcription factor BmAchi/BmVis and *BmUP* promoters with different lengths. (**c**,**d**) Effect of *Bmachi* overexpression on *BmUP* expression in cells. *Bmachi* was over-expressed in BmN cells. Expression of *BmUP* was upregulated in the experimental group. A value of *p* < 0.05 was considered statistically significant (** *p* < 0.01 and *** *p* < 0.001). (**e**,**f**) Effect of *Bmachi* instantaneous interference at the individual level on *BmUP*.

The *BmUP* gene was found to be specifically and abundantly expressed in the silkworm testis (Figure 5b). We hypothesized that this gene might be associated with silkworm sperm development. In order to fully study the regulatory factors upstream of the *BmUP* gene, we performed promoter analysis of the gene. Among the many transcription factors predicted, we identified two interesting transcription factors: *achi/vis*. In Drosophila, *achi/vis* are two highly repetitive homeobox genes. In flies lacking both genes, spermatogenesis is blocked before sperm cell differentiation and the first meiosis [25]. Analysis revealed that these two transcription factors have the same binding sites. We selected the three sites with the highest binding scores (Figure 5a). In order to detect whether silkworm achi/vis transcription factors bind *BmUP*, we constructed an overexpression vector, *Bmachi* (pSL1180-Myc-*Bmachi*)/*Bmvis* (pSL1180-Myc-*Bmvis*). We successfully obtained these two proteins in BmN cells (Figure 5c). With electrophoretic mobility shift assays (EMSAs), we found that the BmAchi protein bound to the three sites of the promoter. However, the BmVis protein did not bind the promoter (Figure 5d,e). We then truncated the promoter according to the three binding sites and cloned the truncated promoters into a dual luciferase reporter vector. By conducting dual luciferase reporter experiments, we found that the length of 1939 bp (including the third binding site) had the strongest promoter activity (Figure 6a,b). Subsequently, we demonstrated that the BmAchi transcription factor specifically binds the third binding site of the *BmUP* promoter through cold competition and super-shift gel migration experiments (Figure 5f,g).

In order to further determine whether BmAchi regulates *BmUP*, we first transfected overexpression vector pSL1180-*Bmachi* and control vector pSL1180-*EGFP* into BmN cells. We found that the expression of *Bmachi* was significantly higher in the experimental group than the control group by qPCR analysis, thus indicating that *Bmachi* was successfully overexpressed in BmN cells (Figure 6c). Simultaneously, we detected the expression of *BmUP*, which was found to be significantly higher in the experimental group than the control group (Figure 6d). This result indicated that BmAchi promotes the transcription of *BmUP*.

We then verified this result at the individual level. In order to perform this verification, we synthesized the double-stranded *Bmachi* RNA in vitro and injected it into D9L individuals to elicit instantaneous interference. Two days after the injection, the testes of the interfering individuals were collected to extract RNA. After conducting qPCR analysis, we found that the expression of *Bmachi* decreased significantly, and instantaneous interference was successful (Figure 6e). We also analyzed expressions of the *BmUP* gene in the experimental group and found that that they were significantly lower than that in the control group (Figure 6f). This result further confirmed the effect of the BmAchi transcription factor in promoting *BmUP* expression.

## 4. Discussion

In the Drosophila sex determination pathway, the *dsx* gene is a key sex determinant and acts as a switch gene. Tra/Tra2 is the main splicing factor that regulates the *dsx* gene and produces different splicing patterns between males and females, and these two splicing factors are also regulated by the upstream *sxl* gene. Active SXL protein is present in females, in which it acts on the downstream *tra* gene and produces active Tra protein. The Tra and Tra2 proteins together act on the *dsx* gene, thereby producing a female splicing mode in females but not males [23,26]. The sex determination mechanism in Bombyx mori differs from that in Drosophila, because the Tra protein is absent in *Bombyx mori*. Analysis indicated that Tra and Tra2 in Drosophila belong to a class of SR enriched proteins. As a splicing factor in fly sex regulation, these proteins regulate *dsx* gene for male and female specific splicing.

SR proteins are a class of serine/arginine-rich proteins involved in constitutive and alternative splicing of precursor RNA [27]. Due to the low molecular weight of the Drosophila Tra protein and its enrichment in arginine and serine, we screened a series of SR proteins from NCBI and finally identified the BmUP protein. We studied the temporal and spatial expression of the *BmUP* gene and found that it is specifically and primarily expressed in the testis. Therefore, we speculate that this gene may be involved in testis development in male silkworms. In this study, with the CRISPR/Cas9 system, we first obtained *BmUP* knockout individuals. We then observed abnormal phenotypes in positive individuals and found that the male knockout external genitalia showed incompletely developed holding organ-like tissues. For further confirmation, we used a piggyBac vector to overexpress the *BmUP* gene in silkworms. In OV-BmUP female and male silkworms, the *BmUP* gene was significantly upregulated at both the mRNA and protein levels. We observed the male and female internal and external genitalia of overexpressing individuals and found that the females’ internal and external genitalia were not significantly different from those in wild-type silkworms. Interestingly, we observed similar phenotypes in the internal and external genitalia of male individuals. The male external genitalia proliferated with excess tissue, the internal genitalia developed in a disordered manner and the sizes of the left and right testes differed. We speculate that this phenotype might have been due to changes in the expression of *BmUP* gene indirectly affecting pathways regulating silkworm genital development. By studying the location of BmUP in the cell, we demonstrated that BmUP is located in the nucleus.

In order to clarify the upstream regulatory mechanism of the *BmUP* gene, we fully analyzed the *BmUP* promoter and found that it binds a series of transcription factors of the Hox gene family, such as *Abd-B*, *Ubx* and *cad*. Due to the fact that the *BmUP* gene is specifically expressed in the testis, *achi* is a transcription factor that regulates spermatogenesis, and *vis* is expressed only in the testis, we selected these two transcription factors for further research. In *Drosophila*, the TG-interacting factor DmAchi/DmVis acts as a transcriptional activator during spermatogenesis [28,29]. In silkworms, the function of *Bmachi* is similar to that of *Dmvis* in *Drosophila*, and both significantly affect sperm differentiation [30]. By conducting dual luciferase reporter experiments, we found that the promoter containing three binding sites had the strongest activity, and the results of EMSA experiments showed that the BmAchi transcription factor specifically binds the *BmUP* promoter. In order to further verify the regulatory effect of BmAchi on *BmUP*, we examined *Bmachi* overexpression; in this context, the expression of *BmUP* increased significantly, whereas interferences with *Bmachi* caused the expression of *BmUP* to significantly decrease. Therefore, BmAchi plays a role in promoting the expression of *BmUP*. Our findings indicate that *BmUP* participates in silkworm gonad development, thus providing new clues for studying the effects of SR proteins on silkworm sex regulation.

## Figures and Tables

**Table 1:** List of primer sequences used in this study.

Primer Name	Primer Sequences	Purpose
sgRNA1-F	5′GGGCAACTATCCCGACCATG3′	gene editing
sgRNA1-R	5′CATGGTCGGGATAGTTGCCC3′	gene editing
sgRNA2-F	5′AACGGTCAGCAGGAGTGCAT3′	gene editing
sgRNA2-R	5′ATGCACTCCTGCTGACCGTTC3′	gene editing
BmUP-F	5′ATGAAGTGTCATTTTGACGTTATTT3′	RT-PCR
BmUP-R	5′TTAAACTGCTTGAATGAATCCC3′	RT-PCR
JC-BmUP-F	5′TCAAATTAATGGATGGATATGCGTA3′	PCR
JC-BmUP-R	5′AAGGGGGTCAAAATCATGTTCAAAG3′	PCR
Qpcr-BmUP-F	5′GAGAACGGTCAGCAGGAGTG3′	qPCR
Qpcr-BmUP-R	5′ACCATCGCTTTGAGCCCAGT3′	qPCR
Qpcr-BmAchi-F	5′CTCTGGAGTCGTTGTCGCTT3′	qPCR
Qpcr-BmAchi-R	5′GTATCTGATGACCGACTGCCA3′	qPCR
Qpcr-BmVis-F	5′GGGCGAACTGGAAAGAG3′	qPCR
Qpcr-BmVis-R	5′TCTACTGAATATATCTGACATCCG3′	qPCR

**Table 2:** Insertion site of Over-BmUP strains.

Transgenic Line	Sequence of Insertion Sites	Chromosome
Over-BmUP	AAAGTCGGTCAACC**TTAA**-piggybac-**TTAA**AAAATATTGATTCC	10

## Data Availability

The data presented in this study are available on request from the corresponding author.

## Abbreviations

achi	Achiya
BmE	Embryonic cells of silkworm
BmN	Ovary cells of silkworm
EMSA EGFP	Electrophoretic mobility shift assay Enhanced green fluorescent protein
QPCR	Quantitative Real-Time PCR
SR	Serine/Arginine-riched protein
vis	Vismay
BCA	Bicinchoninic acid
SDS-PAGE	Sodium dodecyl sulfate polyacrylamide gel electrophoresis
PVDF	Polyvinylidene fluoride
TBST	TBS with Tween-20

## References

[1] Black D.L. (2003). Mechanisms of alternative pre-messenger RNA splicing. Annu. Rev. Biochem..

[2] Black D.L. (2000). Protein diversity from alternative splicing: A challenge for bioinformatics and post-genome biology. Cell.

[3] Wang E.T., Sandberg R., Luo S., Khrebtukova I., Zhang L., Mayr C., Kingsmore S.F., Schroth G.P., Burge C.B. (2008). Alternative isoform regulation in human tissue transcriptomes. Nature.

[4] Will C.L., Luhrmann R. (2001). Spliceosomal UsnRNP biogenesis, structure and function. Curr. Opin. Cell Biol..

[5] Lin S.R., Fu X.D. (2007). SR proteins and related factors in alternative splicing. Altern. Splicing Postgenomic Eraa.

[6] Beier D.H., Carrocci T.J., van der Feltz C., Tretbar U.S., Paulson J.C., Grabowski N., Hoskins A.A. (2019). Dynamics of the DEAD-box ATPase Prp5 RecA-like domains provide a conformational switch during spliceosome assembly. Nucleic Acids Res..

[7] Chou T.B., Zachar Z., Bingham P.M. (1987). Developmental expression of a regulatory gene is programmed at the level of splicing. Embo J..

[8] Boggs R.T., Gragor P., Idrlss S., Belote J.M., Mckeown M. (1987). Regulation of Sexual-Differentiation in D. melanogaster via Alternative Splicing Of RNA from the transformer Gene. Cell.

[9] Amrein H., Gorman M., Nothiger R. (1988). The Sex-Determining Gene tra-2 of Drosophila Encodes a Putative RNA-Binging Protein. Cell.

[10] Fu X.D., Maniatis T. (1992). Isolation of a Complementary-DNA That Encodes the Mammalian Splicing Factor SC35. Science.

[11] Ge H., Zuo P., Manley J.L. (1991). Primary Structure of the Human Splicing Factor ASF Reveals Similarities with Drosophila Regulators. Cell.

[12] Manley J.L., Krainer A.R. (2010). A rational nomenclature for serine/arginine-rich protein splicing factors (SR proteins). Genes Dev..

[13] Kataoka N., Bachorik J.L., Dreyfuss G. (1999). Transportin-SR, a nuclear import receptor for SR proteins. J. Cell Biol..

[14] Valcarcel J., Green M.R. (1996). The SR protein family: Pleiotropic functions in pre-mRNA splicing. Trends Biochem. Sci..

[15] Long J.C., Caceres J.F. (2009). The SR protein family of splicing factors: Master regulators of gene expression. Biochem. J..

[16] Jeong S. (2017). SR Proteins: Binders, Regulators, and Connectors of RNA. Mol. Cells.

[17] Xiao R., Sun Y., Ding J.H., Lin S.R., Rose D.W., Rosenfeld M.G., Fu X.D., Li X. (2007). Splicing regulator SC35 is essential for genomic stability and cell proliferation during mammalian organogenesis. Mol. Cell. Biol..

[18] Li X.L., Wang J., Manley J.L. (2005). Loss of splicing factor ASF/SF2 induces G2 cell cycle arrest and apoptosis, but inhibits internucleosomal DNA fragmentation. Genes Dev..

[19] Zhong X.Y., Wang P., Han J., Rosenfeld M.G., Fu X.D. (2009). SR proteins in vertical integration of gene expression from transcription to RNA processing to translation. Mol. Cell.

[20] Wee C.D., Havens M.A., Jodelka F.M., Hastings M.L. (2014). Targeting SR proteins improves SMN expression in spinal muscular atrophy cells. PLoS ONE.

[21] Fu Y., Wang Y. (2018). SRSF7 knockdown promotes apoptosis of colon and lung cancer cells. Oncol. Lett..

[22] Wan L., Yu W., Shen E., Sun W., Liu Y., Kong J., Wu Y., Han F., Zhang L., Yu T. (2019). SRSF6-regulated alternative splicing that promotes tumour progression offers a therapy target for colorectal cancer. Gut.

[23] Sciabica K.S., Hertel K.J. (2006). The splicing regulators Tra and Tra2 are unusually potent activators of pre-mRNA splicing. Nucleic Acids Res..

[24] Suzuki M.G. (2010). Sex determination: Insights from the silkworm. J. Genet..

[25] Wang Z., Mann R.S. (2003). Requirement for two nearly identical TGIF-related homeobox genes in Drosophila spermatogenesis. Development.

[26] Sun X., Yang H., Sturgill D., Oliver B., Rabinow L., Samson M.L. (2015). Sxl-Dependent, tra/tra2-Independent Alternative Splicing of the Drosophila melanogaster X-Linked Gene found in neurons. G3.

[27] Shepard P.J., Hertel K. (2009). The SR protein family. Genome Biol..

[28] Ayyar S., Jiang J., Collu A., White-Cooper H., White R.A. (2003). Drosophila TGIF is essential for developmentally regulated transcription in spermatogenesis. Development.

[29] Hyman C.A., Bartholin L., Newfeld S.J., Wotton D. (2003). Drosophila TGIF proteins are transcriptional activators. Mol. Cell. Biol..

[30] Zhang P., Cao G., Sheng J., Xue R., Gong C. (2012). BmTGIF, a Bombyx mori homolog of Drosophila DmTGIF, regulates progression of spermatogenesis. PLoS ONE.

